# A randomised controlled trial of meloxicam, a Cox-2 inhibitor, to prevent hepatocellular carcinoma recurrence after initial curative treatment

**DOI:** 10.1007/s12072-016-9704-y

**Published:** 2016-02-04

**Authors:** Yuko Takami, Susumu Eguchi, Masaki Tateishi, Tomoki Ryu, Kazuhiro Mikagi, Yoshiyuki Wada, Hideki Saitsu

**Affiliations:** 1 Department of Hepato-Biliary-Pancreatic Surgery and Clinical Research Institute, National Hospital Organization Kyushu Medical Center, 1-8-1, Jigyohama, Chuo-ku, Fukuoka, 810-8563 Japan; 2 Department of Surgery, Nagasaki University Graduate School of Biomedical Sciences, Nagasaki, Japan

**Keywords:** Hepatocellular carcinoma, Cox-2 inhibitor, Chemoprevention, Recurrence

## Abstract

**Background:**

Because the recurrence rate of hepatocellular carcinoma (HCC) is high, even after curative treatments such as hepatic resection and microwave ablation, chemopreventive agents that can effectively suppress HCC recurrence are required. Cyclooxygenase-2 (Cox-2) was recently found to be overexpressed in HCC. Therefore, Cox-2 inhibitors may offer a chemopreventive therapy for HCC. This randomised controlled trial (RCT) investigated the potential for meloxicam, a clinically used Cox-2 inhibitor, to prevent HCC recurrence after initial curative treatment.

**Methods:**

A total of 232 consecutive patients underwent hepatic resection and/or microwave ablation as initial therapy for HCC at our institute between July 2008 and April 2011. Eight patients were excluded because of poor renal function, history of non-steroidal anti-inflammatory drug-related ulceration, or multiple cancers. The remaining 224 patients were randomised to a control group (*n* = 113) or a meloxicam group (*n* = 111). To patients in the meloxicam group, meloxicam was administered at 15 mg daily (5 mg three times a day) as long as possible. The overall survival (OS) and disease-free survival (DFS) rates were determined.

**Results:**

The 1-, 3-, and 5-year OS rates of the meloxicam group were 95.4, 82.4, and 70.1 %, respectively. Those of the control group were 98.2, 85.1, and 71.5 %, respectively (*p* = 0.9549). The corresponding DFS rates of the meloxicam group were 89.2, 53.9, and 44.0 % and those of control group were 86.5, 57.0, and 43.4 %, respectively (*p* = 0.6722). In the OS and DFS of subsets including patients with hepatitis B or C virus infection, we could not find significant differences between the meloxicam and control groups. However, in the subgroup of analysis of patients without viral hepatitis (NBNC-HCC), significant differences were observed in the DFS between the meloxicam group (1-year DFS, 92.3 %; 3-year DFS, 75.8 %; 5-year DFS, 70.4 %) and control group (1-year DFS, 83.3 %; 3-year DFS, 48.1 %; 5-year DFS, not obtained) (*p* = 0.0211).

**Conclusion:**

Administration of the Cox-2 inhibitor meloxicam may have a possibility to suppress HCC recurrence after initial curative treatments in patients with NBNC-HCC.

## Introduction

Hepatocellular carcinoma (HCC) is globally known as a highly malignant tumor with poor prognosis [1]. A variety of treatment approaches is currently performed for HCC, including liver transplantation [2], hepatic resection [3], local ablative therapies [4, 5], transcatheter arterial chemoembolisation (TACE) [6], particle radiotherapy, and molecularly targeted treatment [7]. In Japan, hepatic resection and local ablative therapies are recommended for small HCCs involving three nodules or less with a diameter of 3 cm at most; however, it frequently recurs after 5 years post treatment. Liver transplantation is an effective treatment option associated with a low recurrence rate and favorable survival. Living donor liver transplantation is predominantly performed in Japan because of the lack of deceased donors, and this limitation inhibits the acceptance of liver transplantation as the treatment of choice.

Since hepatic resection and ablative therapies are still performed as the treatment of choice, measures to prevent recurrence are required. Anti-viral treatments, such as interferons and nucleoside analogues, have been commonly used for the management of hepatitis B or C virus infections [8, 9], respectively. Subsequently, acyclic retinoids [10], branched-chain amino acids, antihypertensives [11], and cyclooxygenase 2 (Cox-2) inhibitors [12] have been clinically introduced.

Cox-2 is known to have effects on cell growth and carcinogenesis, and it plays a role in the development of cancer [13, 14]. It is also well known that Cox-2 inhibitors prevent the development of colorectal cancer [15]. It is also basically clear that Cox-2 is involved in various steps in multiple-step carcinogenesis in HCC [16]. Therefore, we expected that multicentric carcinogenesis in HCC could be prevented by inhibiting these steps.

We previously reported that postoperative treatment with a Cox-2 inhibitor prolonged the survival in patients with recurrent HCC [17].

In the present study, we evaluated the effectiveness of Cox-2 inhibitors as prevention of HCC recurrence, comparing meloxicam, a Cox-2 inhibitor, versus control in patients with HCC who underwent hepatic resection and/or microwave coagulo-necrotic therapy (MCN).

## Materials and methods

Between July 2008 and April 2011, 232 patients with primary HCC underwent hepatic surgery (hepatic resection or MCN) at our institute. Of these, after exclusion of eight patients (renal dysfunction, 2; hepatic dysfunction, 1; history of surgery for pancreatic cancer and aortic aneurysm, 2; ulcer due to NSAIDs, 1; multiple cerebral infarctions, 1; scheduled artery infusion chemotherapy, 1), 224 patients with informed consent from the patient and family were enrolled in the study.

The patients were divided randomly into two groups—111 patients received treatment with meloxicam (meloxicam group), and 113 did not receive meloxicam treatment (control group)—to evaluate disease-free survival (DFS) and cumulative overall survival (OS). To the patients in the meloxicam group, meloxicam was started within 3 weeks after their operation and administered at 15 mg daily (5 mg three times a day) as long as possible.

MCN is a surgical treatment with microwave ablation under laparotomy, small thoracotomy, or laparoscopy. In our previous evaluation of the treatment results of MCN, it was effective for locoregional control of HCC, comparable to hepatic resection. Therefore, we consider MCN as one of the first-choice treatments for HCC [4].

In this study, all patients treated with MCN were regarded as being “without vascular invasion.” The reason is as follows: MCN is a loco-regional treatment in which microwave ablation is performed to treat HCC. Needle biopsy is then performed to obtain samples from the center of the tumor for histological examination. As a result, differentiation of HCC can be determined, but histological vascular invasion cannot. Therefore, all patients treated with MCN were regarded as being without vascular invasion.

DFS was defined as the time from the surgery to any next treatment for recurrent HCC diagnosed using routine abdominal ultrasonography, magnetic resonance imaging (MRI) using gadolinium (Gd)-ethoxibenzyl (EOB)-diethylenetriamine pentaacetic acid (DTPA), or contrast-enhanced computed tomography (CT) scan.

Groups were compared using the Mann-Whitney *U* test or *χ*^2^ test. Survival curves were calculated using the Kaplan-Meier method and compared using a log-rank (Mantel-Cox) test. *p* < 0.05 was considered statistically significant for all analyses.

Before starting this randomised control trial, the protocol was reviewed and approved by an independent institutional review board (IRB) in our hospital.

## Results

For the baseline characteristics of the patients, no significant difference in hepatic function and tumor condition was observed between the groups (Table 1).

**Table 1:** Patient characteristics

	Meloxicam (*n* = 111)	Control (*n* = 113)	*p*
Age (range) (years)	69.5 (42–87)	70.7 (48–87)	0.3329
Gender (M:F)	73:38	70:43	0.5520
Etiology (HBV/HCV/BC/NBNC^a^)	10/73/2/26	14/79/2/18	0.5476
Tumor mean size (mm)	29.4 (9.0–90.0)	27.0 (8.8–86.0)	0.2225
No. of tumors	2.3 (1–10)	1.9 (1–7)	0.1085
Albumin (g/dl)	3.9 (2.7–5.0)	3.8 (1.8–4.9)	0.0902
Total Bilirubin (mg/dl)	0.87 (0.2–2.1)	0.85 (0.3–2.1)	0.7822
Prothrombin time (%)	86.2 (12–170)	88.1 (49–137)	0.3931
Child-Pugh class (A/B)	93/18	88/25	0.2617
AFP (ng/ml)	1574 (2.2–70,200)	181 (0–3576)	0.0585
L3 (%)	10.9 (0–87.1)	8.2 (0–88.0)	0.2571
DCP (mAu/ml)	1758 (6–139,994)	1502 (4.4–39,200)	0.8530
Type of treatment (Hr/Hr + MCN/MCN)	23/9/79	15/3/95	0.0619
Differentiation of HCC (well/moderate/poor)	3/82/14	4/89/8	0.5625
Vascular invasion (positive/negative)	17/94	8/105	0.0503

Data are given as median and range

*AFP* serum α-fetoprotein, *L3* lectin-reactive α-fetoprotein, *DCP* plasma des-γ-carboxy-prothrombin, *Hr* hepatic resection, *MCN* microwave coagulo-necrotic therapy

^a^HBsAg positivity, HCV-Ab positivity, and both HBsAg and HCV-Ab negativity are represented as B, C, and NBNC, respectively

**Fig. 1:**
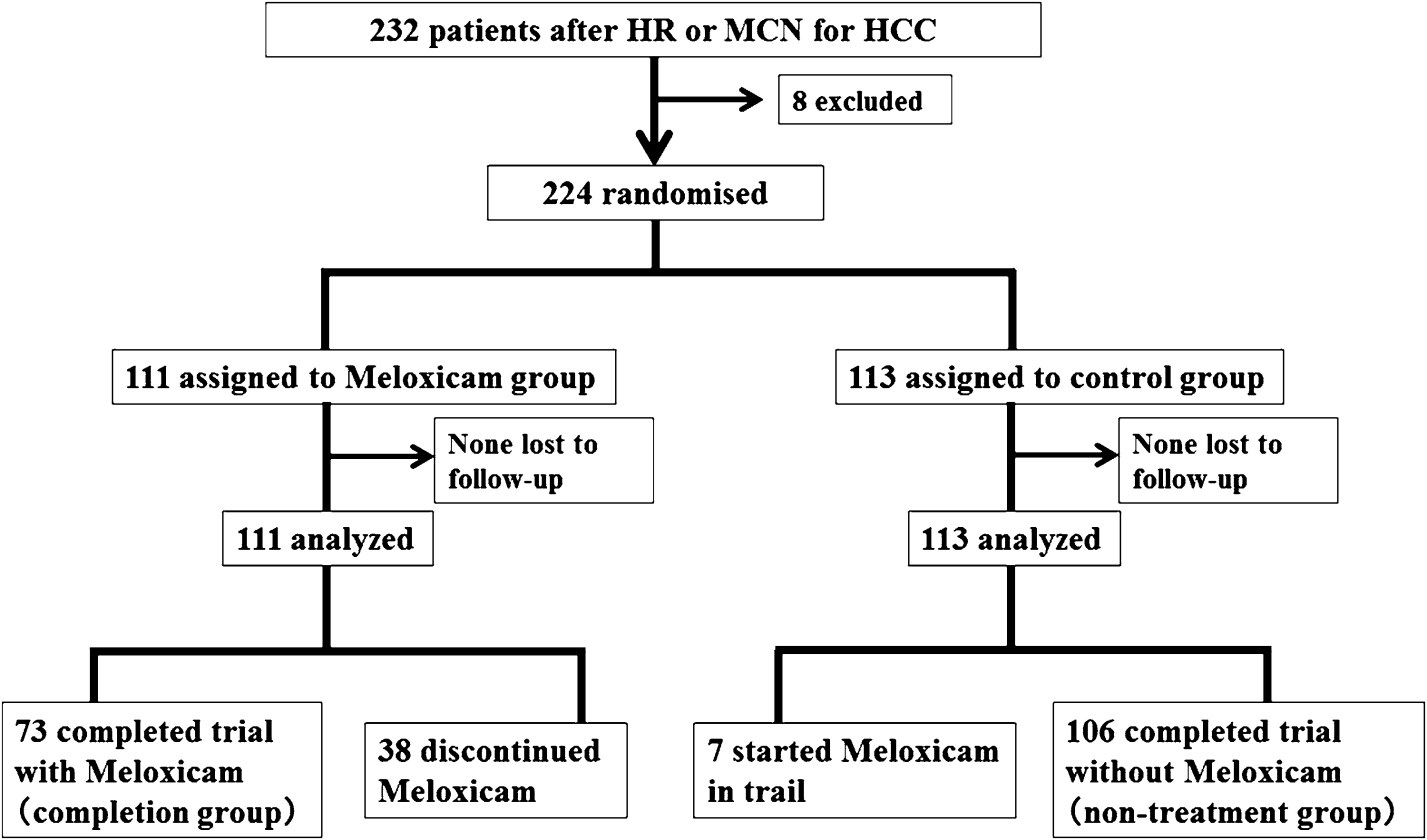
Flow of participants into the study; 224 patients were randomised into a control group or meloxicam group

In the meloxicam group, 73 patients continued meloxicam throughout the study period, and 38 discontinued. Seven patients in the control group started meloxicam in the middle of the study period based on physicians’ discretion, and 106 received no treatment with meloxicam (Fig. 1). The reasons for discontinuation in 38 patients included: reduced renal function (*n* = 12), gastrointestinal tract ulcer (*n* = 5), exacerbation of disease (*n* = 4), prolonged postoperative complications (*n* = 2), cough (*n* = 1), cerebral infarction (*n* = 1), poor adherence (*n* = 5), other cancer break out (*n* = 1), and discontinuation judged by physician (*n* = 7).

**Fig. 2:**
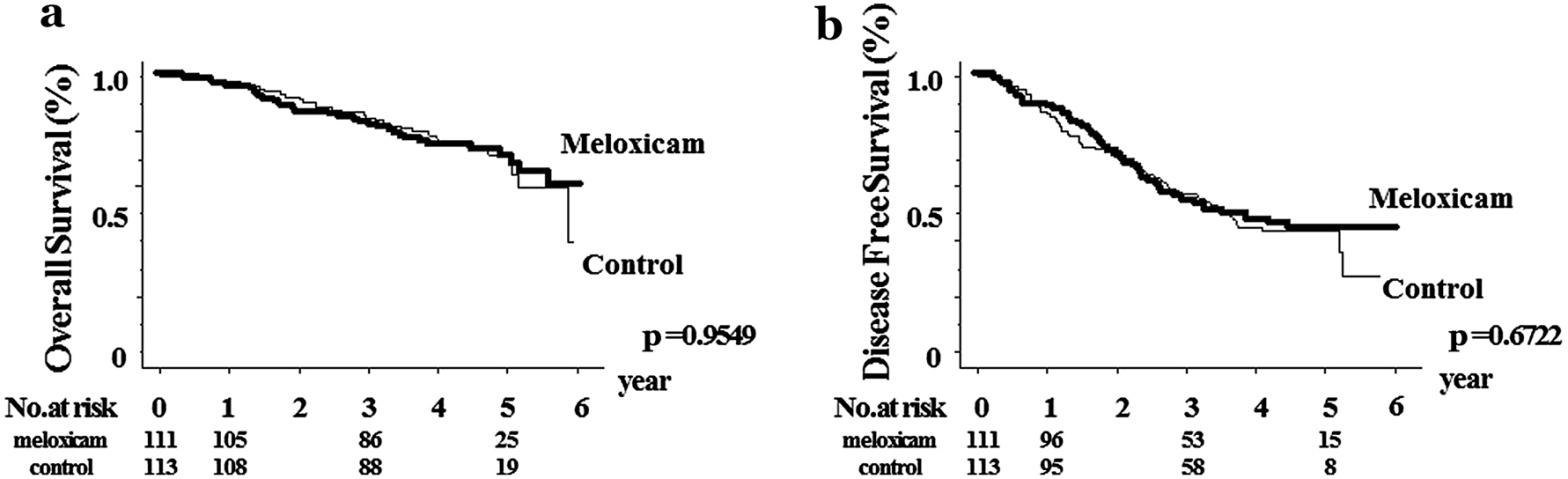
Comparison of the meloxicam group with the control group. Kaplan-Meier estimation of intent-to-treat analyses of overall survival **a** shown, *p* = 0.9549, and disease-free survival **b**, *p* = 0.6722. The number of patients at risk at each time point is shown below the *graphs*

No significant differences in OS were noted between the meloxicam group (1-year OS, 95.4 %; 3-year OS, 82.4 %; 5-year OS, 70.1 %) and control group (1-year OS, 98.2 %; 3-year OS, 85.1 %; 5-year OS, 71.5 %) (*p* = 0.9549). There were no significant differences in DFS between the meloxicam group (1-year DFS, 89.1 %; 3-year DFS, 53.9/ %; 5-year DFS, 44.0 %) and control group (1-year DFS, 86.5 %; 3-year DFS, 57.0 %; 5-year DFS, 43.4 %) (*p* = 0.6722) (Fig. 2).

**Fig. 3:**
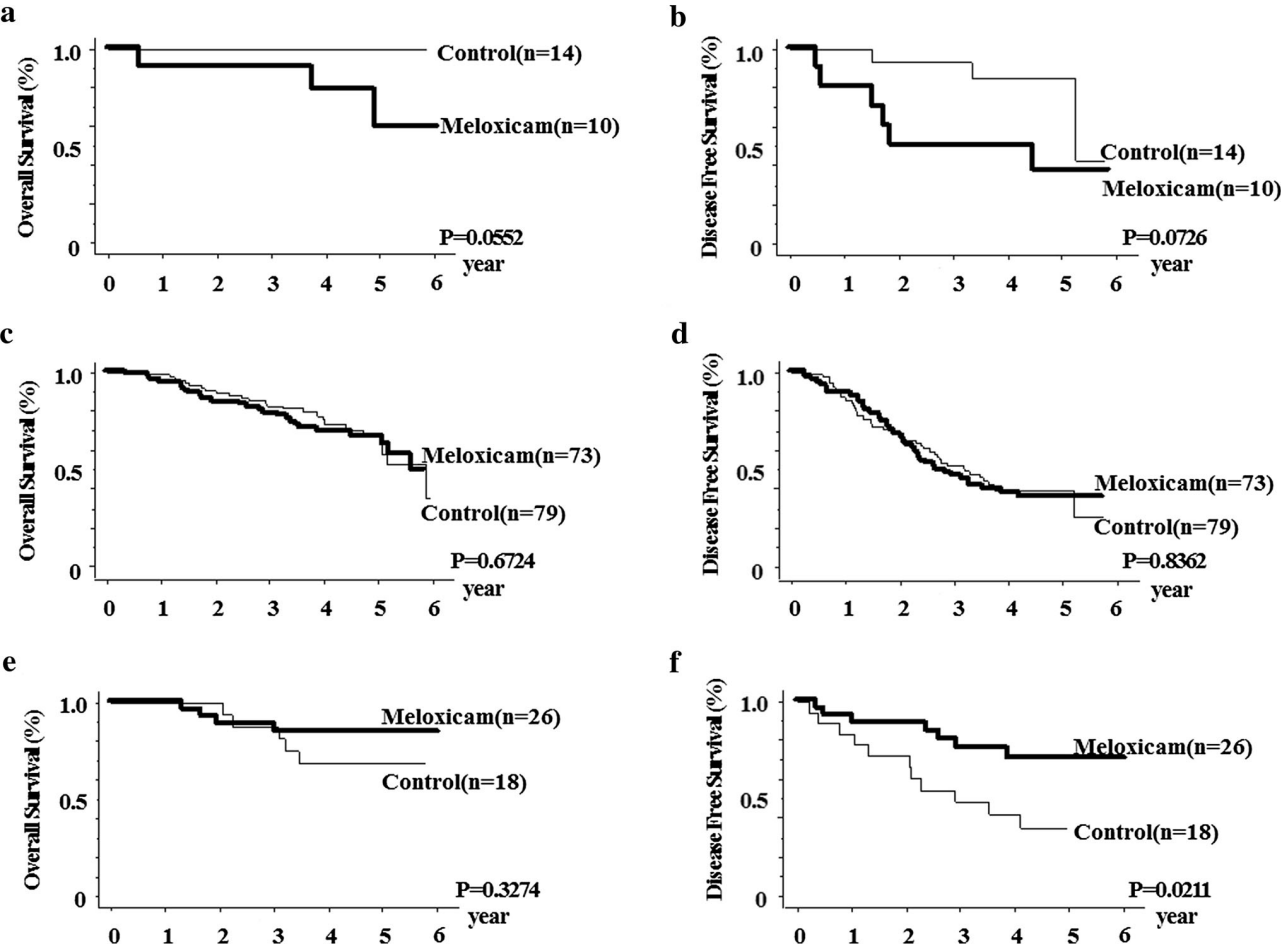
Subgroup analysis. For 24 patients with hepatitis B virus infection, there were no significant differences in OS **a** (*p* = 0.0552) or in DFS **b** (*p* = 0.0726) between the meloxicam group and control group. For patients with hepatitis C virus infection, **c** no significant differences were shown in OS (*p* = 0.6724), or **d** in DFS (*p* = 0.8362) between the two groups. In the remaining 44 patients without hepatitis B or hepatitis C virus infection (NBNC-HCC), no significant differences were noted **e** in OS (*p* = 0.3274), but significant differences were observed **f** in DFS between the meloxicam group and control group (*p* = 0.0211)

For 24 patients with hepatitis B virus infection, there were no significant differences in OS between the meloxicam group (1-year OS, 90.0 %; 3-year OS, 90.0 %; 5-year OS, 59.1 %) and control group (1-year OS, 100 %; 3-year OS, 100 %; 5-year OS, 100 %) (Fig. 3a) and in DFS between the meloxicam group (1-year DFS, 90.0 %; 3-year DFS, 50.0 %; 5-year DFS, 37.5 %) and control group (1-year DFS, 100 %; 3-year DFS, 92.9 %; 5-year DFS, 85.1 %) (*p* = 0.0726) (Fig. 3b). For patients with hepatitis C virus infection, no significant differences were shown in OS between the meloxicam group (1-year OS, 94.4 %; 3-year OS, 78.6 %; 5-year OS, 66.8 %) and control group (1-year OS, 98.7 %; 3-year OS, 82.6 %; 5-year OS, 67.0 %) (*p* = 0.6724) (Fig. 3c) or in DFS between the meloxicam group (1-year DFS, 88.9 %; 3-year DFS, 46.3 %; 5-year DFS, 35.5 %) and control group (1-year DFS, 85.7 %; 3-year DFS, 52.2 %; 5-year DFS, 38.8 %) (*p* = 0.8362) (Fig. 3d). The baseline parameters including the viral condition of patients with hepatitis B or C infection are shown in Table 2.

**Table 2:** Baseline parameters of patients with hepatitis B or hepatitis C

	Meloxicam	Control	*p*
Hepatitis B			
No. of patients	10	14	
Age (years)	56.4 (42–75)	65.9 (50–83)	0.0383*
Internal use of nucleoside analogue (yes/no)	8/2	5/9	0.0318
Detect of HBV-DNA (negative/positive)	3/7	9/5	0.2563
HBV-DNA level in each positive patient (log copies/ml)	5.46	5.12	0.7820
Child-Pugh class (A/B)^a^	6/4	14/0	0.0095
Hepatitis C			
No. of patients	73	79	
Age (years)	71.0 (51–87)	71.1 (48–87)	0.9259
SVR (yes/no)	8/65	10/69	0.7695
HCV-RNA (log IU/ml)	5.96	5.36	0.2375
No. of tumors^b^	2.61 (1–10)	2.01 (1–7)	0.0442

Ages are given as median and range

*SVR* sustained virological response

^a^In the patients with hepatitis B, there were no significant differences between the two groups in the other parameters shown in Table 1

^b^In the patients with hepatitis C, there were no significant differences between the two groups in the other parameters shown in Table 1

In the remaining 44 patients without hepatitis B or hepatitis C virus infection (NBNC-HCC), no significant differences were noted in OS between the meloxicam group (*n* = 26; 1-year OS, 100 %; 3-year OS, 88.5 %; 5-year OS, 84.4 %) and control group (*n* = 18; 1-year OS, 100 %; 3-year OS, 87.5 %; 5-year OS, 68.8 %) (*p* = 0.3274) (Fig. 3e), but significant differences were observed in DFS between the meloxicam group (1-year DFS, 92.3 %; 3-year DFS, 75.8 %; 5-year DFS, 70.4 %) and control group (1-year DFS, 83.3 %; 3-year DFS, 48.1 %; 5-year DFS, not obtained) (*p* = 0.0211) (Fig. 3f). Among the baseline demographic and preoperative parameters of NBNC-HCC patients, no significant difference in patient and tumor conditions was observed between the groups (Table 3).

**Table 3:** Baseline demographic and preoperative parameters of patients without hepatitis B or hepatitis C virus infection

	Meloxicam (*n* = 26)	Control (*n* = 18)	*p*
Age (range) (years)	71.1 (50–85)	72.4 (56–85)	0.6274
Gender (M:F)	21:5	14:4	0.8089
Alcohol intake (daily/chance)	17/9	11/7	0.7723
Diabetis mellitus (±)	17/9	12/6	0.9297
Albumin (g/dl)	4.1 (3.1–4.8)	3.8 (3.0–4.8)	0.0511
Total bilirubin (mg/dl)	0.75 (0.1–1.3)	0.81 (0.3–1.8)	0.5560
Serum AST (IU/l)	36.6 (20–70)	32.2 (20–66)	0.2714
Serum ALT (IU/l)	33.3 (14–87)	26.3 (8–75)	0.0655
Platelet count (10^4^/μl)	17.2 (7.6–30.5)	15.6 (7.1–33.6)	0.4306
Prothrombin time (%)	90.0 (58–118)	88.8 (63–124)	0.7743

Data are given as median and range

## Discussion

Hepatic resection and ablative therapies are the treatment of choice for HCC, and measures to reduce recurrence are needed. The major preventive approach to recurrence is to treat the hepatitis virus infection, which is the main cause of HCC. Ikeda et al. reported that interferon treatment suppressed tumor recurrence after surgery in patients with hepatitis C virus infection, with 80 % reduction when sustained virological response was achieved [8]. In addition, Chen et al. suggested that the viral load was significantly associated with HCC in patients with hepatitis B virus infection, and treatment with nucleoside analogues to reduce the viral load suppresses the occurrence of HCC [9].

Various agents such as acyclic retinoids, branched-chain amino acids [11, 18], antihypertensives such as angiotensin-converting-enzyme inhibitors [19], and Cox-2 inhibitors have been clinically introduced. Chemical stimuli including cytokines and growth factors upregulate the expression of Cox-2, and inflammation induces Cox-2 to mediate increased synthesis of prostaglandin E2 (PGE2) and prostacyclin (PGI2) [13]. PGE2 and PGI2 elicit inflammatory responses by increased vascular permeability (PGE2), vasodilation, and pain sensitivity (PGE2 and PGI2). Furthermore, Cox-2 is revealed to mediate the regulation of cell growth, motility, adhesion, and apoptotic suppression [14]. Increased expression of Cox-2 promotes tumor proliferation, and inhibition of the Cox-2 pathway suppresses carcinogenesis, suggesting Cox-2 may contribute to the development of cancer [20].

Selective COX-2 inhibitors are expected to be effective in clinical use as anti-cancer agents [12]. In addition to prevention of colorectal cancer [15], the association between Cox-2 expression and carcinogenesis of HCC has been reported in many basic and clinical studies [21, 22]. Selective inhibition of Cox-2 is also expected to show activity for cancer prevention [16, 23]. The Cox-2 level in non-cancerous lesions increased from normal liver to chronic hepatitis to cirrhosis, and such expression of Cox-2 may be involved in the postoperative recurrence of HCC [24]. In addition, the expression level of Cox-2 varies in accordance with the degree of differentiation, which suggests Cox-2 may play a role in the early stage of HCC carcinogenesis [25].

Based on the findings from these studies, we administered meloxicam to recurrent small tumors after HCC surgery in a period before the tumor became indicated for retreatment. Treatment with meloxicam in 236 patients with postoperative recurrence of HCC in our institute resulted in a response rate of 9.7 % and disease control rate of 41.8 %. Meloxicam was found to control approximately 40 % of HCC recurrence. Mean time to progression in patients who achieved disease control (complete response, partial response, and stable disease) was 432 days, showing significant prolonged time to retreatment compared with patients with progressive disease. This finding suggested Cox-2 inhibitors could restrict the progression of HCC [17].

Following these clinical data, we conducted a randomised controlled study to consider adjuvant therapy of HCC surgery with or without meloxicam. Unexpectedly, patients with vascular invasion tended to be more common in the meloxicam group. In addition, with regard to differentiation, poorly differentiated HCC tended to be slightly more common in the meloxicam group. Anyway, we could not identify the efficacy of meloxicam in preventing the recurrence of HCC in the study.

We then performed analysis in a subgroup of patients with hepatitis C virus infection. A previous study reported overexpressed Cox-2 might be a prognostic factor in patients with HCC with hepatitis C virus infection [26]. However, in this study we could not demonstrate the preventive efficacy of meloxicam.

Based on the finding that overexpression of Cox-2 in non-cancerous lesions indicates recurrence of HCC in patients with hepatitis B virus-related cirrhosis [27], we performed another analysis in patients with hepatitis B virus infection, which could not show the efficacy of meloxicam. When the two groups were compared in HCC patients with HBV, the patients in the meloxicam group were of younger age, and treatment with nucleoside analogues during surgery was more often observed in the meloxicam group (*p* = 0.0318). In this subanalysis of patients with HBV, unfortunately, there were significant differences in the use of nucleoside analogues and underlying liver function between the two groups, because 224 consecutive patients were randomly assigned to the meloxicam group or the control group.

However, the proportion of patients whose HBV-DNA was reduced to below the limit of detection at the time of the surgery tended to be higher in the control group (*p* = 0.2563), and underlying liver function was significantly better in the control group (*p* = 0.0095).

In the meloxicam group, most patients started dosing immediately prior to the start of treatment, and it was speculated that there was insufficient time to reduce the viral load before the treatment. Based on this, lowering the HBV-DNA to below the limit of detection for a long period of time is considered important in preventing subsequent hepatic cancer.

In patients with neither hepatitis B nor hepatitis C (NBNC-HCC), DFS was significantly higher in the meloxicam group compared with the control group, which suggests these patients may be a population in which the preventive efficacy of meloxicam is achieved. For the baseline demographic and preoperative parameters of these patients, no significant difference was observed between the two groups. However, this population consisted of patients with various backgrounds including alcohol-induced hepatitis and non-alcoholic steatohepatitis (Table 3); thus, it is difficult to discuss the mechanism of meloxicam in such patients with the limited data from our study. Further research with a larger sample size with a well-defined background is warranted. We consider that, as the cause of the ineffectiveness of meloxicam in patients with HCC involving hepatitis B or hepatitis C virus infection, the impact of the hepatitis virus on HCC was potent enough to counteract that of meloxicam.

In 34 % of patients who could not continue meloxicam, the most common cause was the decrease in renal function. A recent study reported that long-term treatment with NSAIDs in patients with rheumatoid arthritis did not affect renal function much [28]. In the present study, we prescribed discontinuing meloxicam when the eGFR fell below 45 ml/min/1.73 m^2^, and 12 patients in the meloxicam group discontinued meloxicam after approximately 2 years (mean, 772 days). A decrease in renal function may influence the prognosis of HCC, which requires management of edema and ascites, and thus should be considered as an important adverse effect that requires careful caution. Five patients discontinued meloxicam because they developed ulcers. Gastrointestinal mucosal damage is one of potential adverse reactions of NSAID treatment [29], and a long-term proton pump inhibitor (PPI) is commonly used for such damage [30]. In our study, we could prevent ulcer formation with the use of PPI; however, long-term treatment with meloxicam and PPI is difficult considering the health insurance coverage in Japan. Therefore, in order to establish the preventive effect of Cox-2 inhibitors for HCC, management of renal function and ulcer formation is required as well as identification of the patient population.

## Conclusion

Our results suggested the possibility of a preventive effect of postoperative treatment with meloxicam in patients with HCC not involving hepatitis B or hepatitis C virus infection (NBNC-HCC). Further research is highly warranted to determine the effect of meloxicam on various types of HCC in a larger sample size.

## List of abbreviations

HCC: Hepatocellular carcinoma
MCN: Microwave coagulo-necrotic therapy
Cox-2: Cyclooxygenase-2
